# Fluorescent Dissolved Organic Matter Components as Surrogates for Disinfection Byproduct Formation in Drinking Water: A Critical Review

**DOI:** 10.1021/acsestwater.2c00583

**Published:** 2023-06-12

**Authors:** Elena Fernández-Pascual, Boris Droz, Jean O’Dwyer, Connie O’Driscoll, Emma H. Goslan, Simon Harrison, John Weatherill

**Affiliations:** † School of Biological, Earth and Environmental Sciences, University College Cork, Cork T23 TK30, Ireland; ‡ Environmental Research Institute, University College Cork, Cork T23 XE10, Ireland; § iCRAG Science Foundation Ireland Research Centre in Applied Geosciences, University College Dublin, Dublin D04 V1W8, Ireland; ∥ Ryan Hanley Ltd., Castlebar F23 E400, Ireland; ⊥ Cranfield Water Science Institute, Cranfield University, Cranfield MK43 0AL, United Kingdom

## Abstract

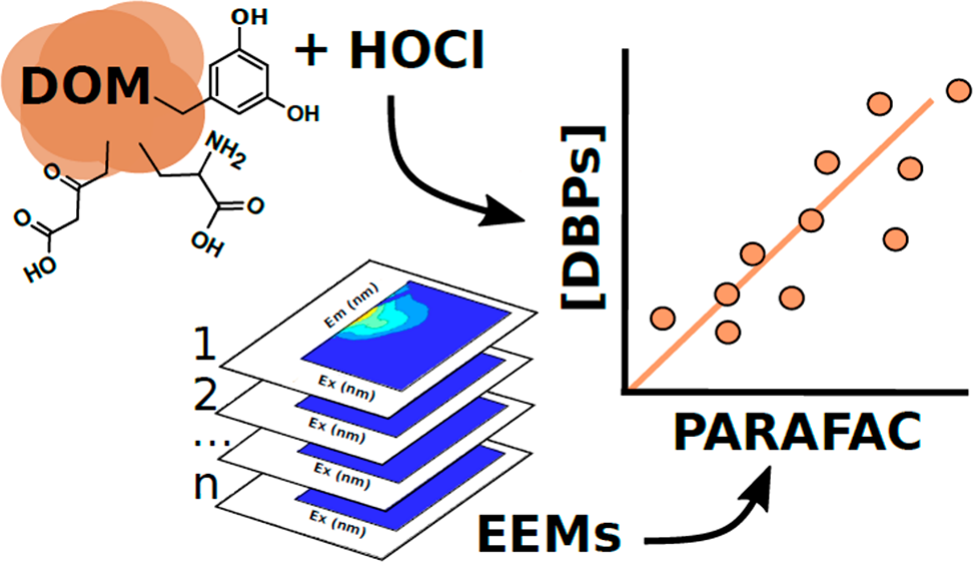

Disinfection byproduct (DBP) formation, prediction, and minimization are critical challenges facing the drinking water treatment industry worldwide where chemical disinfection is required to inactivate pathogenic microorganisms. Fluorescence excitation–emission matrices-parallel factor analysis (EEM-PARAFAC) is used to characterize and quantify fluorescent dissolved organic matter (FDOM) components in aquatic systems and may offer considerable promise as a low-cost optical surrogate for DBP formation in treated drinking waters. However, the global utility of this approach for quantification and prediction of specific DBP classes or species has not been widely explored to date. Hence, this critical review aims to elucidate recurring empirical relationships between common environmental fluorophores (identified by PARAFAC) and DBP concentrations produced during water disinfection. From 45 selected peer-reviewed articles, 218 statistically significant linear relationships (*R*^2^ ≥ 0.5) with one or more DBP classes or species were established. Trihalomethanes (THMs) and haloacetic acids (HAAs), as key regulated classes, were extensively investigated and exhibited strong, recurrent relationships with ubiquitous humic/fulvic-like FDOM components, highlighting their potential as surrogates for carbonaceous DBP formation. Conversely, observed relationships between nitrogenous DBP classes, such as haloacetonitriles (HANs), halonitromethanes (HNMs), and *N*-nitrosamines (NAs), and PARAFAC fluorophores were more ambiguous, but preferential relationships with protein-like components in the case of algal/microbial FDOM sources were noted. This review highlights the challenges of transposing site-specific or FDOM source-specific empirical relationships between PARAFAC component and DBP formation potential to a global model.

## Introduction

The use of chlorine and other chemical disinfection methods (such as ozonation or chloramination) for drinking water disinfection can lead to the unintentional formation of potentially harmful disinfection byproducts (DBPs), through reactions with dissolved organic matter (DOM) precursors present in the raw water source.^1^ Consequently, nine organohalide DBPs including four trihalomethanes (THM4) and five haloacetic acids (HAA5) are regulated by drinking water authorities in the European Union (EU)^2^ and United States (US).^3^ However, in excess of 700 DBPs have been identified to date, the vast majority of which are unregulated with many thoughts of being potentially carcinogenic.^4^ Moreover, a recent study estimated that 32–81% of total organic halogen (TOX) loads, produced during chemical disinfection, are attributable to as yet unidentified DBPs, highlighting the likely importance of new and emerging classes over the coming years.^5^

DOM in freshwaters is composed of a multitude of soluble, reduced organic carbon compounds, which may be derived from autochthonous sources, such as in situ primary production (e.g., algae and microbial biomass), and allochthonous watershed sources, such as leaf litter and soil organic matter leachates^6^ whose hydrological export varies spatially and temporally within river basins around the world.^7^ Humic and fulvic acids (typically originating from allochthonous terrestrial sources) comprise high molecular weight and aromaticity humic substances, which are thought to be important DOM precursors for carbonaceous DBP (C-DBP) classes.^8,9^ In contrast, autochthonous DOM derived from algal and microbial sources, as well as wastewater DOM, may play a role in the formation of nitrogenous DBPs (N-DBPs),^10,11^ which are believed to be potentially more harmful to human health than C-DBPs.^12^ A global increase in terrestrial DOM export is forecast over the coming decades as a consequence of climate and environmental change.^13,14^ Increasing DOM concentrations in raw water sources derived from surface water will pose significant challenges for safe and sustainable production of drinking water, over the coming decades.

**Figure 1:**
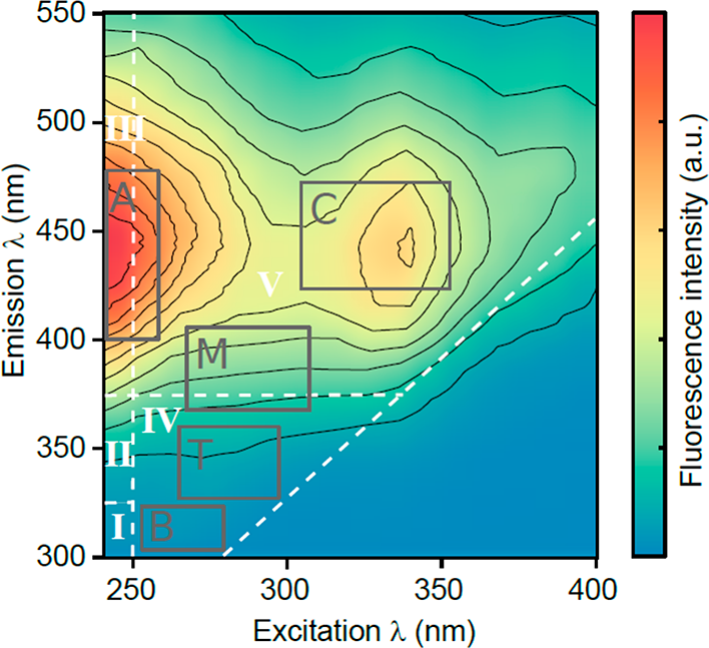
Classical emission–excitation matrix (EEM) of riverine dissolved organic matter showing fluorescence mainly in the fulvic and humic regions (River Bunsheelin, Ireland; 4th July 2021). White dashed lines delimit the five common environmental fluorescence regions.^24^ Gray boxes refer to local wavelength pair regions at which fluorophore maximum intensities are generally “picked”.^23^

Modern advances in fluorescence excitation–emission matrix (EEM) spectroscopy have offered a unique perspective on fluorescent dissolved organic matter (FDOM) characterization and quantification in freshwater environments. Fluorescence spectroscopy is a low-cost, nondestructive, sensitive, and selective technique that can provide critical information on the molecular properties of complex FDOM admixtures.^15^ The technique also offers considerable promise for quantification of intrinsic environmental fluorophores, which may be associated with DBP formation.^16−22^ Various methods have been developed to extract qualitative and quantitative information from EEM spectra such as “peak picking”^23^ and fluorescence regional integration (FRI) (Figure 1).^24^ More recently, principal component analysis (PCA) or parallel factor analysis (PARAFAC) have been applied to reduce the dimensionality of large EEM data sets into a small number of independent components.^25,26^ Whereas PCA decomposes EEMs into components which are not physically meaningful, PARAFAC is capable of “unmixing” complex EEM spectra to resolve the underlying independent fluorophores present and has become the EEM decomposition and interpretation tool of choice. Machine learning (ML) approaches, such as artificial neural networks (ANNs) and self-organizing maps (SOMs) are becoming more available for applications with fluorescence spectroscopy data.^27−29^ ML approaches complement EEM-PARAFAC outputs and offer pathways toward greater automation in classification^30−32^ and regression analysis^33^ of large and complex EEM data sets.

### Scope of the Critical Review

Previous reviews of EEM-PARAFAC have included: a general overview of the technique,^26^ applications in drinking water and wastewater treatment plants,^34,35^ critical evaluation of commonly used fluorescence metrics,^36^ potential pitfalls of oversimplification of EEM interpretations,^37^ application of SOMs for EEM data analysis,^28,38^ new approaches for similarity metrics,^39^ and the practical challenges for continuous, online monitoring applications.^40,41^ However, to date, no article has critically reviewed the potential of ubiquitous environmental fluorophores as low cost optical surrogates for DBP formation potential at a global scale. Hence, the present critical review aims to identify statistically significant and recurrent empirical relationships between FDOM components (identified by PARAFAC) and DBP formation reported in the available literature. Consideration is given to the potential advantages of PARAFAC components for continuous online monitoring applications and early warning detection of DBP formation risk in drinking water, with the overarching goal of protecting consumer health.

## Literature Review

Out of 378 identified articles, 45 were selected for inclusion (hereafter referred to as the “selected articles”) which matched the scope and search criteria of the present study (procedure fully detailed in Supporting Information, Figure S1 and Table S1). Data from the selected articles were extracted into categories such as raw water sources, EEM acquisition procedures, FDOM components, chemical disinfection method, and DBP formation potential parameters (temperature and contact time). Relevant data fields arising from the selected articles are collated in SI, Extracted Data (TXT file).

### PARAFAC Components

PARAFAC components identified in the selected articles were classified according to their wavelength pairs into the five environmental fluorescence regions (following the established nomenclature) and are summarized in Table 1. Statistically distinct emission values (pairwise Wilcoxon test; *p*-value: <0.01) are reported between the fluorophore regions except between humic- and fulvic-like components, which can be considered as one group containing similar chemical structures with different degrees of oxidation, molecular size, and solubility at low pH.^42^ Moreover, according to the carbon mass fraction, fulvic substances are estimated to be more abundant than humic^43^ and exhibit higher fluorescence intensities.^23^

**Table 1:** PARAFAC Components Identified and Classified by Wavelength Pair from the Selected Articles

		range (min–max) λ maximum (nm)^b^			
fluorophore region^a^	*n*	excitation	emission	peak label^a^	environmental sources^c^
humic-like (V)	299 (188)	250–375 (255–410)	300–520	C	Terrestrial or river source containing polyhydroxylated aromatics such as those found in lignin, as well as phenols, hydroquinones, and indoles. Generally insoluble fraction at neutral pH.
fulvic-like (III)	41 (33)	250–265 (238–360)	400–475	A	Terrestrial and marine source of aromatic species, such quinones or other oxidized aromatics. Generally smaller molecules, more polar and soluble than humic-like.
microbial humic-like (V)	55 (33)	250–346 (285–374)	300–459	M	Originally identified in marine environments associated with biodegradation of humic-like or with specific proteins or metabolic byproducts.
tyrosine-like (IV)	103 (49)	250–280 (270–285)	300–328	B	Free or bound amino acids associated with microbial activity, autochthonous source.
tryptophan-like (IV)	135 (58)	250–300 (255–300)	300–390	T	Soluble protein or byproduct associated with microbial activity, autochthonous source.

a Follows the traditional assignment and peak label made elsewhere.^23,24,44,45^ Roman numerals delimit the common environmental fluorescence regions where components were identified (Figure 1). Secondary excitation maxima are shown in parentheses. *n* indicates the number of components described in the selected articles used to calculate the range of excitation maxima with the secondary maxima in parentheses.

b Range of excitation and emission wavelengths were not considered below 240 and 300 nm, respectively, due to potentially deteriorating signal-to-noise ratios.

c Potential environmental sources are described elsewhere.^23,24,46−52^

### DBP Classes

DBP classes have been previously classified as follows:^3^ (i) carbonaceous DBPs (C-DBPs), i.e., trihalomethanes (THMs), haloacetic acids (HAAs), haloketones (HKs), haloacetaldehydes (HALs), halogenated furanones (X-furanones), and iodinated THMs (I-THMs), and (ii) nitrogenous DBPs (N-DBPs), i.e., halonitromethanes (HNMs), haloacetonitriles (HANs), haloacetamides (HAMs), *N*-nitrosamines (NAs), and cyanide (CNX). In total, 41 individual DBP species (4 THMs, 9 HAAs, 2 HKs, 1 HAL, 1 X-furanone, 6 I-THMs, 1 nonclassified C-DBP, 1 HNM, 4 HANs, 2 HAMs, 9 NAs, and 1 CNX) were evaluated.

**Figure 2:**
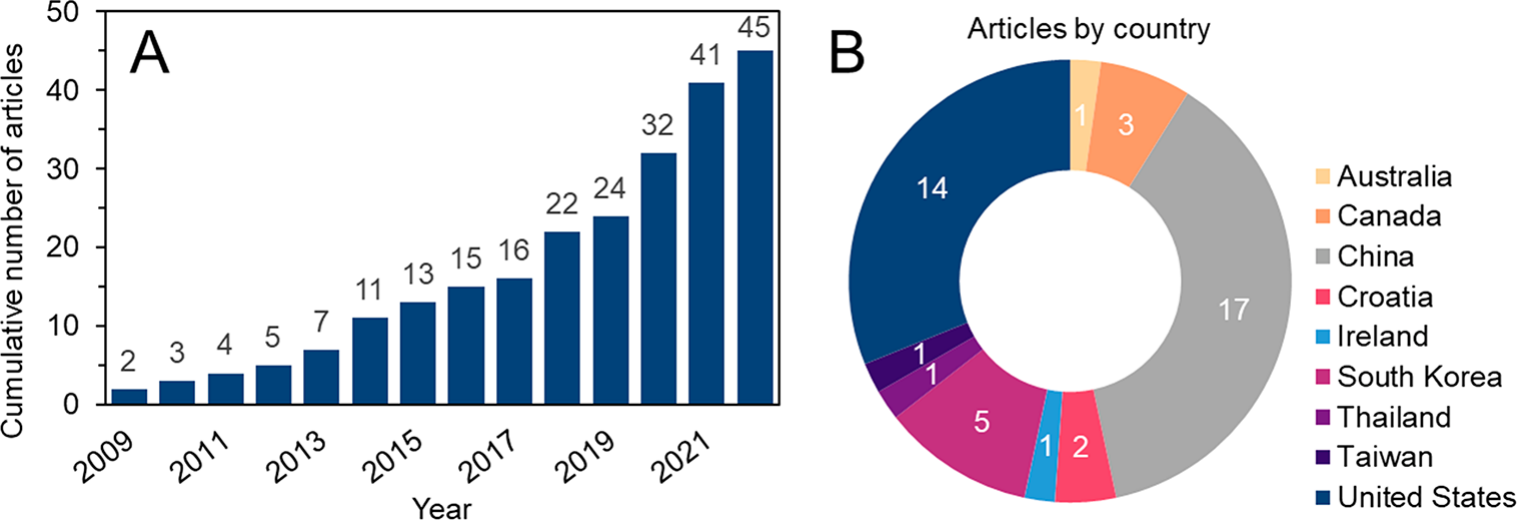
(A) Cumulative number of selected articles (*n*_articles_ = 45) from 2009 to 2022 included in this critical review. The cumulative number is indicated above the bars. (B) Country of origin and number of selected articles published by each country.

The 45 selected articles were published between 2009 and 2022 (Figure 2). Significant growth in the awareness of DBPs and potential associations with EEM-PARAFAC components is noteworthy from 2017 onward (Figure 2). Selected articles focus mainly on drinking water treatment plant optimization or upgrades (47%), tracking the spatiotemporal dynamics of DOM in surface water (15%), and evaluating issues of biofilm algae (17%) or species-specific leaf leachates (9%). Additionally, some articles investigated the photoirradiation impact on DOM (10%) and a comparison of methods of DOM characterization (3%). A large diversity of DOM sources, e.g., surface water (71%), algal/microbial DOM (24%) and leaf leachate (10%), were investigated and are summarized in the SI. Dissolved organic carbon (DOC) concentrations reported within the selected articles ranged from 0.03 to 1,000 mg C L^–1^.

## Experimental Approaches

### EEM-PARAFAC Sample Preparation

PARAFAC methodologies for EEM decomposition have been reviewed elsewhere.^26,53^ Sample preparation methods prior to EEM acquisition included filtration, dilution, and/or pH adjustment of various aqueous matrices. EEM spectra were typically recorded for excitation and emission wavelength pairs higher than 240 and 300 nm, respectively, where shorter wavelengths are thought to be associated with deteriorating signal-to-noise ratios.^37^ Commonly represented PARAFAC toolboxes included DOMFluor^54^ and drEEM,^26^ present in 62% and 17% of the selected articles, respectively, with other toolboxes also noted (detailed in SI, Extracted Data).

### Disinfection Byproduct (DBP) Formation Potential

Two approaches were described in the selected articles to evaluate DBP formation, which are fully reported in SI, Extracted Data. The first and most common approach (employed in 84% of the selected articles) was a collection of composite samples from the water treatment plant with EEM acquisition and DBP formation evaluated under controlled conditions (e.g., pH, temperature, disinfectant dose and contact time).^55^ The second approach, employed in 16% of studies (such as in refs (56 and 57)), involved acquisition of EEMs from the raw water source followed by postchlorination sampling for DBPs at points within the distribution network. This second approach may have some limitations as DBP yield is generally a function of residence time within water distribution networks which varies between 1 and 3 days from chlorination to the consumer tap.^58^ All DBP formation reactions were arrested using an appropriate quenching agent with ascorbic acid being the most popular (SI, Table S2). Ascorbic acid is shown to have good compatibility with a broad spectrum of DBPs including THMs, HAAs, HKs, HALs, and HANs.^59^

## Recurrent Associations between FDOM Components and DBP Formation

**Figure 3:**
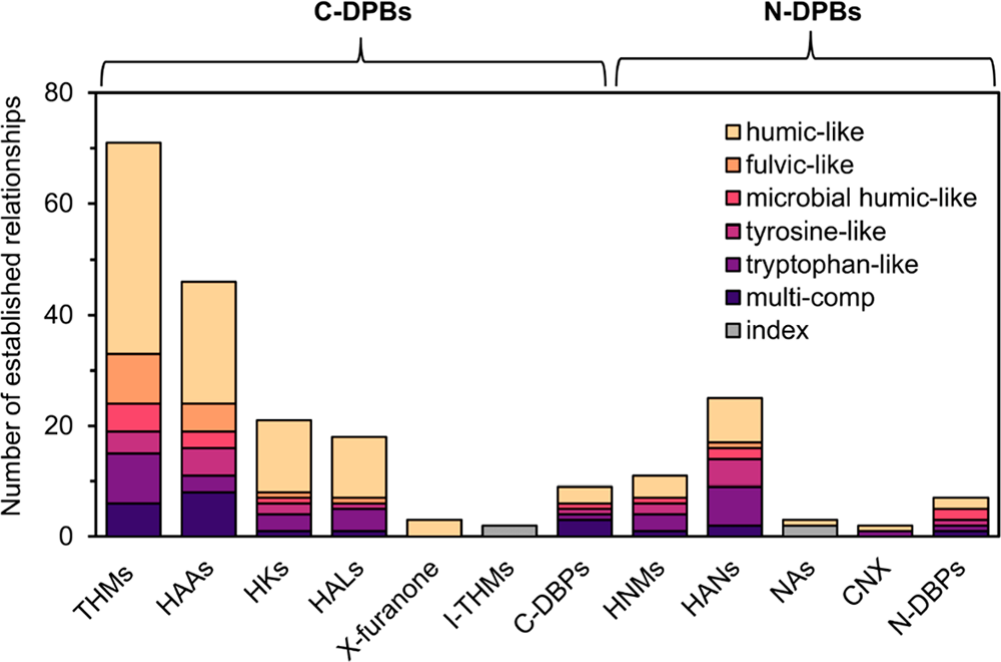
Number of moderate to strong established relationships (*R*^2^ ≥ 0.5) described in the 45 selected articles as a function of DBP class and PARAFAC component (*n*_total_ = 218). “Multicomp” and “index” refer to relationships derived from multiple linear regression models (MLR) or the sum of PARAFAC components and PARAFAC component indices (e.g., humic-like divided by tryptophan-like, respectively).

From the 45 selected articles, 218 empirical relationships between PARAFAC component intensities and DBP concentrations (Figure 3) were observed within the selected articles, of which 135 had strong linear relationships (e.g., Pearson correlation coefficient, *R*^2^ ≥ 0.7; SI, Extracted Data) and 83 had moderate linear relationships (e.g., *R*^2^ ≥ 0.5–0.7), hereafter referred to as “established relationships”. Overall, a larger proportion of relationships between C-DBP classes (Figure 3) and humic/fulvic-like components compared to other FDOM components were found in the selected articles as follows: 74%, 67%, 71%, and 76% for THMs, HAAs, HKs, and HALs, respectively. In contrast, a similar proportion of relationships between N-DBP classes and humic/fulvic-like versus protein-like, i.e., tyrosine/tryptophan-like components, were observed. Direct comparisons between the selected articles were not always straightforward as EEM-PARAFAC and DBP formation potential methodologies were not uniform and model performance parameters rarely reported. Most established relationships were generally derived from the entire data set using the Pearson correlation coefficient (*R*^2^) which constituted a satisfactory metric of the variance for a single model but not a transferable metric to compare two or more models.^60^ Error metrics, e.g., root-mean-square error, slope, and intercept, which may aid in intercomparison between models and for model performance evaluation were never discussed in the selected articles. In addition, models trained on a random subset of the data set where the remaining data are used to cross-validate the model performance were absent in the selected articles. Therefore, some caution is warranted in the transferability of the reported established relationships to predict DBP formation, where occasionally strong relationships may be coincidental^61^ or site-specific for a single DOM source.^62,63^

### THM and HAA Classes

THM and HAA classes reported in the selected articles contained 11 regulated DBPs (SI, Table S3) including four THMs (THM4), i.e., trichloro- (TCM), bromodichloro- (BDCM), dibromochloro- (DBCM), and tribromomethane (TBM), and five HAAs (HAA5), i.e., monochloro-, dichloro-, trichloro-, monobromo-, and dibromoacetic acid, with four additional unregulated HAAs, i.e., bromochloro-, bromodichloro-, dibromochloro-, and tribromoacetic acid.^64,65^ It is noteworthy that trichloromethane was the dominant DBP, with up to 92% of THM4 and up to 47% of the TOX load,^66^ observed from chlorination/chloramination in drinking water treatment plants when bromide concentrations were low.^16,56,67−70^ THMs and HAAs were investigated in 88% and 51% of the selected articles (SI, Table S4), respectively, which together accounted for 53% of the total established linear relationships with PARAFAC components (Figure 3).

Strong, recurring relationships between humic- and fulvic-like FDOM components and THM and HAA formation (e.g., *R*^2^ ≥ 0.7) indicate these ubiquitous environmental fluorophores may be significantly associated with THM and HAA formation globally. On the other hand, established relationships between THMs and HAAs with UV absorbance at 254 nm (*A*_254_), especially specific ultraviolet absorbance at 254 nm (SUVA_254_), were made in 35% of cases, for both classes. SUVA_254_ is directly proportional to the aromaticity of DOM,^71^ which is consistent with aromatic DOM as a precursor for THM and HAA formation.^8^ In addition, the relationship between THMs and HAAs with UV absorbance demonstrate that, although PARAFAC components are a stronger surrogate for prediction of THMs and HAAs, UV absorbance may be a technically simpler approach and should be considered depending on the purpose and extent of prediction desired.^55^

Humic and fulvic acid compounds form a complex mixture of aromatic and aliphatic hydrocarbon structures with functional groups including amide, carboxyl, hydroxyl, and ketone,^6^ which were postulated within the selected articles as important precursors of THMs and HAAs,^8^ originating in surface water from fresh plant or leaf litter leachate (Table 1). This observation is consistent with electrophilic attack on carbonyl functional groups such as aldehydes, ketones, and carboxylic acids, which is thought to be one of the major pathways in the production of THMs and HAAs.^72^ In addition, changes in EEM spectra may indicate a change in DOM molecular structure, where a decrease in fluorescence intensity over all components during chlorination was widely observed.^17,22,73,74^ Decreases in fluorescence intensity tend to support a hypothesis that degradation of aromatic DOM accompanied by the release of DBPs may occur simultaneously with chlorination.^74,75^ Finally, to account for the additive and linear contribution of PARAFAC components, multiple linear regression (MLR) models or the sum of PARAFAC components showed substantial relationship improvement in comparison to individual PARAFAC component models.^18,73,76−78^ This suggests that several DOM compounds with different fluorescence regions may be associated with precursors for THMs and HAAs.

### Brominated (Br-DBPs) and Iodinated DBPs (I-DBPs)

Br-DBPs and I-DBPs are formed when bromide (Br^–^) and iodide (I^–^) ions are present in source waters. These species react quickly with hypochlorous acid (HOCl) to form hypobromous (HOBr) or hypoiodous acid (HOI), which may further react with DOM under the same pathway as HOCl.^79,80^ However, HOBr reacts typically up to 3 orders of magnitude quicker than HOCl and has a very high reactivity with phenol functional groups.^79^ Therefore, the incorporation yield of Br^–^ into THMs is around 50% compared to 5–10% for Cl^–^.^81^ From Table 2, it can be seen that there are a large number of moderate to strong relationships between humic- and fulvic-like fluorophores and Br-DBP formation. Interestingly, the observed ratio between the variation of bromide and PARAFAC components (ΔBr/ΔC_PARAFAC_) before and after chlorination exhibit a strong relationship with Br-DBP formation potential, e.g., Br-THMs, Br-HAAs, and Br-HANs. Conversely, weak relationships have been observed with an individual PARAFAC component model in the same study.^82^ Similar to Br-DBP formation, PARAFAC component indices (Table 2) derive a stronger linear relationship with I-DBP formation compared to individual PARAFAC components.^62^ This observation may be consistent to some extent with the variation of the reactivity between HOI and the nature and location of aromatic substituents on the phenolic moieties.^83^

**Table 2:** Established Relationships between DBP Species and PARAFAC Components per Disinfection Method and DBP Class^a^

DBP formation potential^b^	PARAFAC component(s)^c^	correlation coefficients (*R*^2^)^d^	ref
Chlorine – Trihalomethanes (THMs)			
trichloromethane (TCM)	hum/ful/m-hum/tyr/tryp/multi	≥0.71**(5), ≥0.52**(3)/≥0.70** (2)/0.70*, ≥0.61*(2)/0.61*/0.82*, 0.57**/≥0.77*(2)	(16, 18, 68, 69, 73, 84, 85)
dibromochloromethane (DBCM)	tyr/tryp	0.54**/0.50**	(86)
TCM, bromodichloromethane (BDCM), DBCM	hum/ful	0.96*/0.92*	(87)
TCM, BDCM, DBCM, tribromomethane	hum/ful/m-hum/tyr/tryp/multi	≥0.71**(15), ≥0.52*(9)/≥0.70*(4)/0.82*, 0.66*/0.95, 0.67**/0.84**, ≥0.52 (5)/0.95, 0.69**	(51, 63, 67, 69, 84, 88−95)
Chlorine Dioxide – THMs			
TCM	hum	≥0.92 (4)	(96)
Ozone – THMs			
TCM	multi	0.91**	(97)
BDCM	multi	0.90**	(97)
Chlorine – Haloacetic Acids (HAAs)			
dichloroacetic acid (DCAA)	hum/ful/multi	0.58**/≥0.53**(2)/0.61**	(18, 98)
trichloroacetic acid (TCAA)	hum/ful/multi	0.73**, 0.52**/0.68**(2)/0.70**	(18, 98)
monochloroacetic acid (MCAA), DCAA, TCAA	hum/m-hum/tyr/tryp/multi	≥0.73*(4), ≥0.51 (2)/0.64*/0.71*/0.87*/0.91*	(73, 93)
DCAA, TCAA, bromochloroacetic acid (BCAA)	hum/ful	0.93*/0.89*	(87)
DCAA, TCAA, dibromoacetic acid (DBAA)	hum/multi	0.72**, 0.67**/≥0.69**(2)	(78)
MCAA, DCAA, TCAA, monobromoacetic acid (MBAA), DBAA	hum/tyr/tryp/multi	≥0.81**(4), ≥0.55 (2)/≥0.82(2)/0.74/≥0.83 (2)	(17, 76, 88, 92, 94)
MCAA, DCAA, TCAA, BCAA, MBAA, DBAA	hum/m-hum/tryp	≥0.71**(2), 0.65*/≥0.56*(2)/0.54*	(91, 99)
MCAA, DCAA, TCAA, MBAA, DBAA, tribromoacetic acid, BCAA, bromodichloroacetic acid, dibromochloroacetic acid	hum	0.71	(92)
Ozone – HAAs			
DCAA	multi	0.90**	(97)
UV + Chlorine – HAAs			
DCAA	tyr	0.57**	(100)
TCAA	tyr	0.64**	(100)
Chlorine – Haloketones (HKs)			
1,1-dichloro-2-propanone (DCP)	hum/tyr/tryp	≥0.86**(2), ≥0.52**/0.54**/0.60**	(101, 102)
1,1,1-trichloro-2-propanone (TCP)	hum/ful/tryp	≥0.71*(5), ≥0.56 (3)/0.81*/0.72**	(87, 93, 102)
DCP, TCP	hum/m-hum/tyr/tryp/multi	≥0.72*(2)/0.71*/0.71*/0.76*/0.86*	(63, 73)
Chlorine – Haloacetaldehydes (HALs)			
2,2,2-trichloroethane-1,1-diol (chloral hydrate)	hum/ful/tyr/multi	≥0.72*(6), ≥0.50**(2)/0.52**/0.77**/0.78**	(51, 93, 94, 98)
UV + Chlorine – HALs			
chloral hydrate	hum/tryp	≥0.96 (4)/≥0.95 (4)	(22)
Chlorine – Halogenated Furanones (X-Furanones)			
4-chloro-3-dichloromethyl-2*H*-furan-5-one	hum	≥0.70*(3)	(103)
Monochloramine – Iodinated DBPs (I-DBPs)			
dichloroiodomethane, bromochloroiodomethane, dibromoiodomethane, chlorodiiodomethane, bromodiiodomethane, triodomethane	index	≥0.96 (2)	(62)
Chlorine – Carbonaceous Disinfection Byproducts (C-DBPs)			
monochloroacetic acid, dichloroacetic acid, trichloroacetic acid, TCM, DCP, TCP	hum/m-hum/tyr/tryp/multi	0.78*/0.70**/0.72*/0.88*/0.92*	(73)
TCM, BDCM, DBCM, TBM, dichloroacetic acid, trichloroacetic acid, dibromoacetic acid	hum/multi	≥0.62**(2)/≥0.62**(2)	(78)
Chlorine – Halonitromethanes (HNMs)			
trichloronitromethane (chloropicrin)	hum/m-hum/tyr/tryp/multi	≥0.73*(4)/0.75*/≥0.71*(2)/≥0.80*(2), 0.65**/0.85*	(51, 63, 73, 89)
Chlorine – Haloacetonitriles (HANs)			
bromochloroacetonitrile	hum/m-hum/tyr/tryp	0.76**/0.79**/0.59**/0.57**	(86)
dichloroacetonitrile	hum/tyr/tryp	≥0.83*(3), 0.60/0.52**/0.66**	(93, 102)
dichloroacetonitrile, bromochloroacetonitrile	hum/ful	0.90*/0.85*	(87)
dichloroacetonitrile, trichloroacetonitrile, bromochloroacetonitrile, dibromoacetonitrile	hum/tyr/tryp/multi	0.55/≥0.6 (2)/≥0.85**(3), 0.64/0.73**	(51, 63, 89, 94)
dichloroacetonitrile, trichloroacetonitrile	hum/m-hum/tyr/tryp/multi	0.56*/0.64*/0.64*/0.78*/0.80*	(73)
Monochloramine – *N*-Nitrosamines (NAs)			
*N*-nitrosodiphenylamine (NDPhA)	hum/index	0.6/0.6	(104)
*N*-nitrosomorpholine	index	0.55	(104)
UV + Monochloramine – Cyanide (CNX)			
cyanogen chloride	hum/tryp	0.91/0.79	(22)
Chlorine – Nitrogenous Disinfection Byproducts (N-DBPs)			
dichloroacetonitrile, bromochloroacetonitrile	hum/m-hum	0.50**/0.62**	(86)
trichloronitromethane, trichloroacetonitrile, dichloroacetonitrile	hum/m-hum/tyr/tryp/multi	0.77*/0.80*/0.78*/0.86*/0.94*	(73)

a This table is a summary of data extracted from the 45 selected articles (complete data fields are provided in SI, Extracted Data as a TXT file). Established (linear) relationships for similar DBP species and disinfection methods are separated by a forward slash.

b Reported DBP and chemical disinfection method employed.

c Identified PARAFAC components classified by excitation–emission wavelength pairs into common environmental fluorescence regions (reported in Table 1), where hum, ful, m-hum, tyr, and tryp refer to humic, fulvic, microbial-humic, tyrosine, and tryptophan-like fluorophores, respectively. In addition, “multi” and “index” refer to relationships derived using multiple linear regression models or sum of PARAFAC components and component indices, e.g., humic-like divide by tryptophan-like, respectively.

d Strong linear relationships (*R*^2^ ≥ 0.7) and moderate linear relationships (*R*^2^ ≥ 0.5–0.7) between similar DBPs species and PARAFAC components are differentiated by a comma. Number of established relationships are expressed in parentheses; * and ** indicate *p*-values of ≤0.05 and ≤0.01, respectively. In the case of several significant relationships, only the highest *p*-value is reported.

### Other C-DBPs

Other C-DBPs such as HKs and HALs were investigated in 33% and 20% of the selected articles, respectively (SI, Table S4). Like THMs and HAAs, they exhibited many moderate and strong relationships with humic/fulvic-like components (Figure 3). PARAFAC components were particularly suitable as a surrogate for these DBPs as 75% and 63% of the investigated relationships exhibited moderate or strong relationships (Table 2) for HKs and HALs, respectively. Overall, these other C-DBP classes share similar relationships to THMs and HAAs with humic/fulvic-like fluorophores described above.

### N-DBP Classes

N-DBP classes were not as well investigated in the 45 selected articles as C-DBPs. N-DBP classes were represented by HNMs, HANs, NAs, and CNX in 29%, 50%, 5%, and 2% of the selected articles (Figure 3), respectively. Despite the low number of articles for N-DBP classes, noteworthy observations could still be ascertained. Overall, the total number of established relationships with humic/fulvic-like PARAFAC components versus protein-like fluorophores were almost identical across the N-DBP classes (Figure 3 and Table 2). In addition, none of the selected articles reported the PARAFAC component associated with wastewater or nutrient-enriched waters identified in a previous study (λ_ex_/λ_em_: 350/428 nm).^105^ This observation is surprising because humic/fulvic DOM generally has a low organic nitrogen mass ratio (e.g., <5% N/C), compared to wastewaters or algal-derived DOM, which can have up to 20% N/C.^106^ In addition, protein-like components which contain a high amino acid and *N*-organic compound content^52^ may both serve preferentially as N-DBP formation precursors.^10^ In the context of identified algal/microbial DOM sources, 72% of the successfully established relationships for chlorination were between protein-like components and HNM and HAN formation potential,^73,89,107,108^ which is in agreement with the N-DBP formation precursors described above. In contrast, other DOM sources, e.g., leaf leachate and natural soil/water organic matter, showed no preferential relationship with humic/fulvic-like or protein-like components and the formation of N-DBPs. These observations support the conclusion that proteinaceous DOM is not necessarily the main precursor of N-DBPs and that their formation pathways may not always involve a similar amine precursor.^10,11^ Some N-DBP formation pathways may involve inorganic nitrogen species (e.g., NH_4^+^_, NO_2_^–^ or NO_3_^–^) or chloramine as a nitrogen source which reacts with humic/fulvic-like DOM to produce N-DBPs,^10^ but this cannot be concluded from PARAFAC modeling alone.

## Discussion and Research Outlook

### Environmental Implications

**Figure 4:**
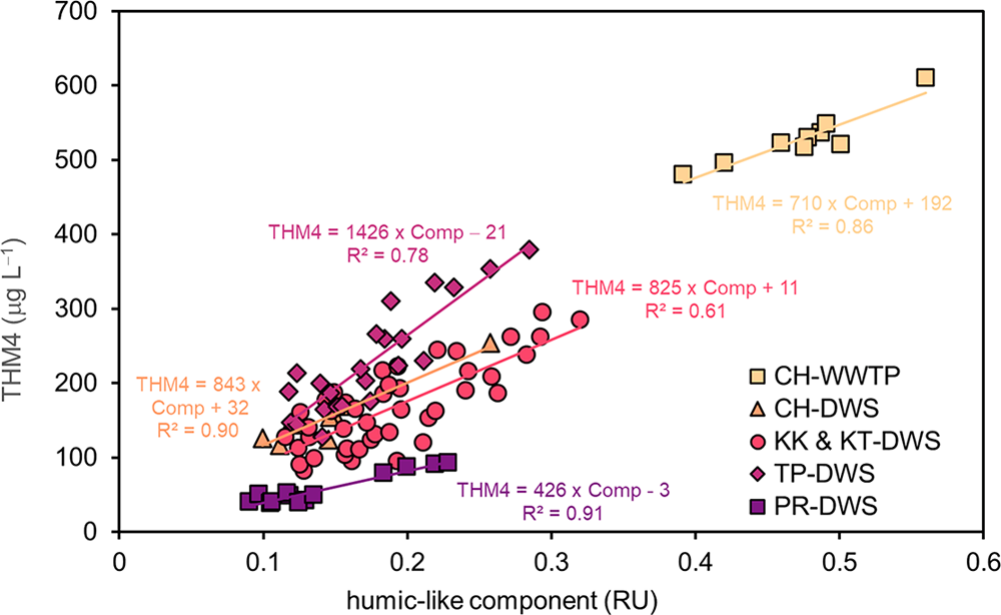
Composite of observed relationships between humic-like PARAFAC components (Comp) and total trihalomethane (THM4) formation in a subset of the selected studies. Samples originated from several drinking water sources (DWS) and a wastewater treatment plant (WWTP). The similarity of slopes across all five independent study regions and DOM sources is noteworthy. CH refers to Chapel Hill, North Carolina, US.^63^ KK, KT, and TP refer to Kotha, Tapra, and Khon Kaen University in Khon Kaen, Thailand, respectively.^84^ PR refers to Pyeongchang river in Korea.^99^ Data published in quinine sulfate units (QSUs) were transformed into Raman units (RUs) using the relationship of Lawaetz et al.^109^ It should be noted that there is some uncertainty with the RU value positioning along the *x*-axis as instrument-specific conversion factors were not available.^110^

Relationships between THMs and SUVA_254_ are well established (see section on THM and HAA classes).^16,68,73,77^ From the findings of this review, a majority of the strong, recurring empirical relationships were observed between carbonaceous DBPs and humic/fulvic-like fluorophores (Figure 4) across the selected articles (Table 2) (SI, Extracted Data). Given that EEM-PARAFAC components represent the small number of independent underlying fluorophores present in raw water FDOM admixtures, they offer a much more selective surrogate for quantitative prediction of DBP formation potential in comparison to classical optical parameters such as UV absorbance or fluorescence peak picking.^16,17,51,73,85,89,96^ However, some articles were noted where PARAFAC components improve only marginally (Δ*R*^2^ ≤ 0.1) the prediction of DBP formation potential.^21,68,77,90^ Furthermore, linear models developed with one specific DOM source and/or location with an associated fluorophore intensity are not necessarily transposable to other environments (Figure 4).^62,63^

One possible explanation is that DOM originating from different sources may exhibit contrasting chemical composition but yield similar fluorescence intensities.^111^ Additional analytical methods, such as high resolution-mass spectroscopy (HR-MS),^112^ size-exclusion chromatography,^113^ and size fractionation,^16^ may help to better constrain the molecular basis of FDOM components across different environmental sources. For example, HR-MS undertaken on environmental DOM samples has demonstrated that PARAFAC components can describe up to 59% of nontarget DOM species,^114^ which highlights the unique capabilities of EEM-PARAFAC as a low-cost tool in DOM characterization. ML algorithms (such as ANNs) already in use for fluorescence data^33,115^ may help to improve the success of DBP formation prediction using PARAFAC components. ML approaches offer distinct advantages by taking into account nonlinear relationships, interaction between variables, and diverse variable types, e.g., continuous and discrete, and do not rely on predetermined physical-based rules or assumptions on a given data set.^60^ However, the successful deployment of ML relies on having sufficient contrasting data to train and properly validate an algorithm capable of recognizing subtle differences in FDOM composition.^116^ This finding highlights a clear need for community sharing of raw EEM spectra globally to be able to perform unified analysis under the same workflow, in a similar manner to the online repository “OpenFluor” for PARAFAC models.^117^ Within the 45 selected articles, no raw EEM spectra were shared. While it is not generally practical to include such large data volumes in publication supporting information, online repositories such as acs.figshare^118^ and Zenodo^119^ are readily available which embrace FAIR principles of data sharing^120^ and are advocated further.

### Implications for Continuous Online Monitoring

Two approaches for continuous online monitoring applications have recently been explored: (i) selection of specific excitation–emission wavelength pairs from PARAFAC analysis and monitoring of intensities to predict online DBP formation with relatively inexpensive in situ UV light-emitting diodes (LED) fluorescence sensors;^93,121,122^ (ii) acquisition of full EEM spectra using a laboratory fluorometer connected to the source water via an in situ fiber-optic sensor in combination with SOMs.^123^ New, faster PARAFAC algorithms now available^124,125^ will allow continuous online data processing where EEMs can acquired at near-real time frequencies. Currently, method (i) is the most cost-effective method where UV-LEDs offer a narrow spectral bandwidth centered on a specific wavelength pair and are commercially available for humic-like (λ_ex_/λ_em_: 320–370 ± 15/450–490 ± 30 nm) and tryptophan-like (λ_ex_/λ_em_: 270–285 ± 30/340–350 ± 30 nm) regions. An exhaustive list of current commercially available sensors and overview of key practical aspects in the use of UV-LEDs have been reviewed elsewhere.^41,126^ Custom made UV-LED systems have also been explored by some authors,^121,127^ and the field is expected to grow with the rapid development of new UV-LEDs becoming available on the market.^128^ In addition, there are several practical challenges reviewed by Henderson^40^ for continuous online fluorescence applications in surface water. For example, some distortion of the fluorescence signal will be observed under high suspended sediment loads or rapid changes in temperature, which may require additional in situ instrumentation, postprocessing, and validation with a laboratory fluorometer for continuous monitoring applications.^129,130^

## Conclusions

To the best of our knowledge, the present study represents the first critical review to collate and extract recurring associations between observed PARAFAC components and the formation of specific DBP classes during chemical disinfection. From the 45 selected articles, we found 218 statistically significant linear relationships between the formation of 10 DBP classes and observed PARAFAC components; of these, 135 were strong (*R*^2^ ≥ 0.7). From the findings of this review, humic- and fulvic-like fluorophores, typically originating from allochthonous watershed sources demonstrate considerable potential as low-cost fluorescence surrogates (*R*^2^ ≥ 0.7) for the formation of multiple C-DBP classes including THMs, HAAs, HALs, and HKs. In contrast, the formation of potentially more harmful N-DBP classes exhibited strong linear relationships across all fluorophore regions. However, where algal or microbial autochthonous DOM sources were present, protein-like fluorophores (e.g., tryptophan-like) alone show strong relationships with N-DBP formation. Here, assigning specific components as surrogates for N-DBP formation is more challenging and source-specific than humic- and fulvic-like fluorophores for C-DBPs. Relationships derived from multiple linear regression or sums of PARAFAC components tended to show stronger predictive capability compared to an individual component model. Predicting DBP formation from FDOM components during drinking water treatment presents new opportunities for continuous online monitoring applications where treatment process operation and optimization are informed by source water FDOM composition with a view toward minimizing harmful concentrations of DBPs in consumer tap water.

## Supporting Information Available

Further information on the literature review (article selection criteria, classification of PARAFAC components, scope of selected articles) (PDF)
Extracted data from the 45 selected articles used in this review (TXT file format with a header directly readable in an R environment); metadata file (PDF) explaining the extracted data fields and coding (ZIP)
