# Supplementary material for: Fluorescent Dissolved Organic Matter Components as Surrogates for Disinfection Byproduct Formation in Drinking Water: A Critical Review
Source: ACS ES T Water. 2023 Jun 12;3(8):1997–2008. doi: 10.1021/acsestwater.2c00583 (PMC10425960; doi:10.1021/acsestwater.2c00583)
Supplement: Supplementary file 1 — ew2c00583_si_001.pdf [file ew2c00583_si_001.pdf]

Fluorescent Dissolved Organic Matter Components as Surrogates for Disinfection Byproduct Formation in Drinking Water: A Critical Review

Elena Fernández-Pascual, ¥, †,‡ Boris Droz, ¥, †,‡ Jean O'Dwyer, †,‡,§ Connie O'Driscoll, ‖ Emma H. Goslan ⊥, Simon Harrison †,‡ John Weatherill* †,‡,§*

† School of Biological, Earth and Environmental Sciences, University College Cork, Cork, T23 TK30, Ireland.

‡ Environmental Research Institute, University College Cork, Cork, T23 XE10, Ireland.

§ iCRAG Science Foundation Ireland Research Centre in Applied Geosciences, University College Dublin, Dublin, D04 V1W8, Ireland

‖ Ryan Hanley Ltd., Castlebar, F23 E400, Ireland.

⊥ Cranfield Water Science Institute, Cranfield University, Cranfield, MK43 0AL, UK.

¥ These authors contributed equally to the publication.

* Corresponding author. e-mail: john.weatherill@ucc.ie and bodroz@bluewin.ch

phone: +353 (0)21 490 4578

Supporting Information

11 Pages

1 Figure

4 Tables

ABBREVIATION LIST

ANN	artificial neural networks
BDCM	bromodichloromethane
C-DBPs	carbonaceous DBPs
CNX	cyanide
DBCM	dibromochloromethane
DBPs	disinfection byproducts
DOC	dissolved organic carbon
DOM	dissolved organic matter
EEM	excitation−emission matrix
FAIR	findable, accessible, interoperable, reusable
FRI	fluorescence regional integration
HAAs	haloacetic acids
HALs	haloacetaldehydes
HAMs	haloacetamide
HANs	haloacetonitriles
HKs	haloketones
HNMs	halonitromethanes
HOBr	hypobromous acid
HOCl	hypochlorous acid
HOI	hypoiodous acid
HR-MS	high-resolution mass spectroscopy
I-THMs	iodinated THMs
LED	light-emitting diodes
NAs	*N*-nitrosamines
N-DBPs	nitrogenous DBPs
NDMA	*N*-nitrosodimethylamine
PARAFAC	parallel factor analysis
PCA	principal component analysis
SD	standard deviation
SEC	size exclusion chromatography
SOMs	self-organizing maps
TBM	tribromomethane
TCM	trichloromethane
THMs	trihalomethanes
UFC	uniform formation condition
UV	ultraviolet
X-furanones	halogenated furanones

LITERATURE REVIEW

Article selection criteria

A four step search criteria was applied to select relevant articles (hereafter referred to as the 'selected articles') for critical review as follow: 1) identification, 2) screening; 3) eligibility, and 4) inclusion (Figure S1). Several databases were consulted, primarily Web of Science, Scopus and Mendeley. The search was made with a combination of relevant keywords including "EEM", "PARAFAC", "components" and "disinfection byproducts". Google Scholar and ResearchGate were used as supplementary search engines. In addition, the references of the selected articles were screened to further identify articles that could not be found through the initial database screening.

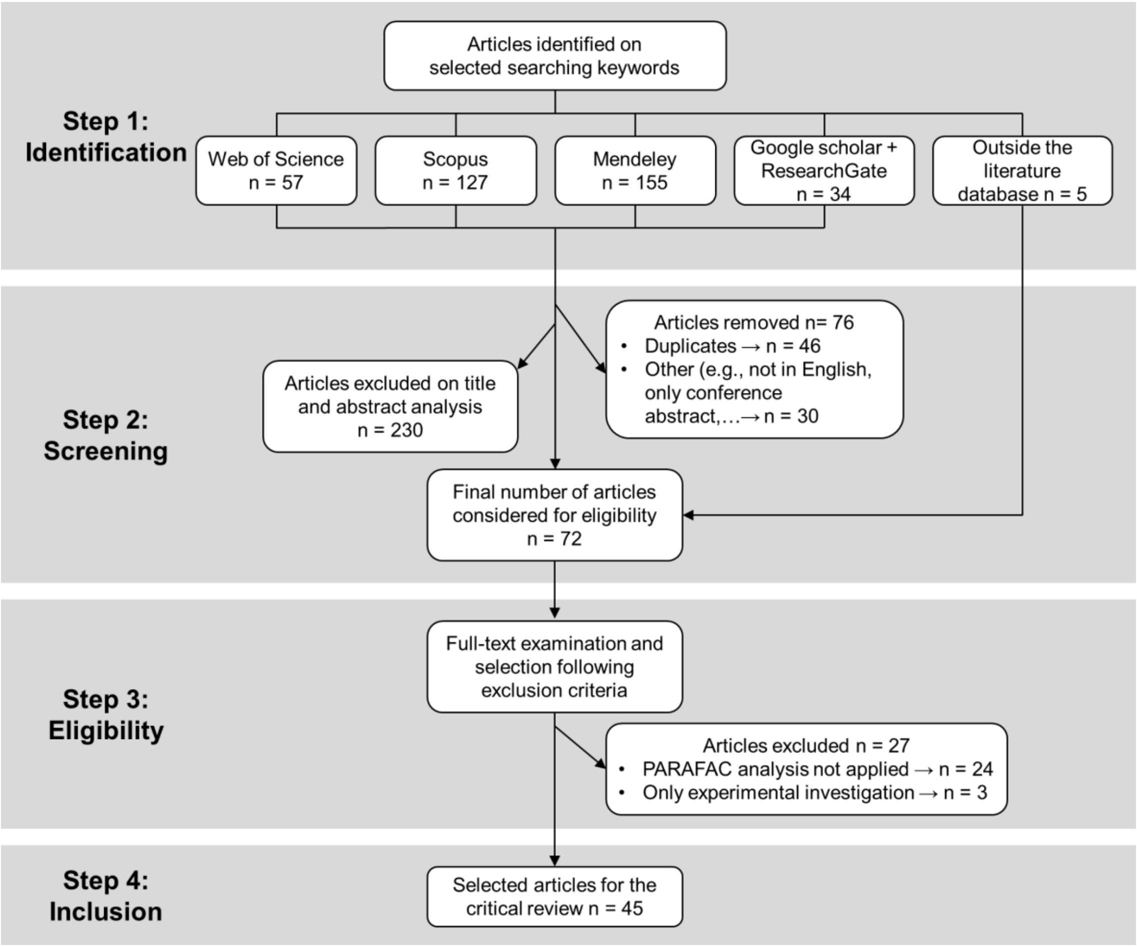

Figure S1. Workflow of the steps and criteria use to select the 45 articles.

All relevant articles published until September 2022 were retrieved from databases during the identification step. Inclusion/exclusion criteria, used to determine the eligibility of the articles are detailed in Table S1. During the screening step, duplicates, records not written in English or full articles not readily available were excluded. Likewise, articles were excluded if they only focused on PARAFAC modelling or on measurement of DBPs alone. Articles where the research question was outside the scope of the present review were also excluded. Five additional articles were identified outside the literature database by screening the references of the selected articles. In the eligibility step, articles that used fluorescence EEM spectroscopy but where PARAFAC modelling was not applied, were also excluded. Articles only reporting laboratory experiments not employing environmental samples were not considered further.

Table S1. Inclusion/Exclusion Criteria Used to Determine the Eligibility of the Articles.

**Step**	Inclusion criteria	Exclusion criteria
**1**	Original research	Not written in English and not complete information (e.g., conference abstract)
**2**	Investigate raw or post-treated drinking water and/or other environmental samples (e.g., leaf leachates)	Purely experimental studies not involving environmental samples
**3**	Evaluates DBP formation potential and applies PARAFAC analysis	Evaluates DBP formation potential without PARAFAC analysis. Apply other EEM analytical methods (e.g., peak picking, FI, PCA)
**4**	Implements PARAFAC analysis to characterize DOM or its removal efficiency by various water treatment options	Implement PARAFAC analysis for a purpose other than DOM characterization or its removal efficiency by various water treatment options
**5**	Investigate pattern or relationship between DBPs formation potential and PARAFAC components	DBPs and PARAFAC relationships (e.g., linear, polynomial) were not investigated in the article

Classification of PARAFAC components

As part of the present study, all PARAFAC components identified within selected articles were extracted and compiled into TXT file (SI, extracted data) which may be found free of charge at the ACS website. To facilitate categorization and comparisons between fluorophore regions and PARAFAC components, data were classified following the nomenclature provided in previous articles and defined in Table 1.[1-3] Following this approach, a total of five fluorophore region were established[4] following the original classification made by Coble.[1] The classification covers the most common environmental fluorescence regions and was established as follow: humic-like (peak ‘C’), fulvic-like (peak ‘A’), microbial humic-like (peak ‘M’) and

protein-like. Other peaks, such as those derived from photobleaching (peak H), phytoplankton (peak 'N') and soil fulvic acid (peaks 'D' and 'E') have only very seldom been observed in source waters[3, 5] and hence were considered to be outside the scope of current review.

Reference to 'protein-like' fluorophore type only in the selected articles was further refined to discriminate between tryptophan-like (peak 'T' region; n = 113) and tyrosine-like (peak 'B' region; n = 80) fluorophores. The threshold to distinguish between tryptophan-like and tyrosine-like components was made on the 75th percentile of the emission wavelength range (min–max) which were significantly different, e.g., 340–356 nm ($\bar{X}$ = 350 nm) and 306–324 nm ($\bar{X}$ = 320 nm) for tryptophan and tyrosine-like regions, respectively. The excitation wavelength range on the 75th percentile did not show a significant difference, with overlapping ranges of 235–255 nm ($\bar{X}$ = 245 nm) and 230–275 nm ($\bar{X}$ = 230 nm) for tryptophan-like and tyrosine-like respectively.

Scope of selected articles

A yearly cumulative increase in the number of articles matching the review criteria was observed (Figure 2). The selected article studies were carried out in nine different countries, in order of importance, China ($n_{articles}$ = 17), United States ($n_{articles}$ =14), South Korea ($n_{articles}$ = 5), Canada ($n_{articles}$ = 3), Croatia ($n_{articles}$ = 2), Australia ($n_{articles}$ = 1), Ireland ($n_{articles}$ = 1), Taiwan ($n_{articles}$ =1) and Thailand ($n_{articles}$ = 1; Figure 2). **Water sources** investigated were mainly from surface water ($n_{articles}$ = 29), followed by groundwater ($n_{articles}$ = 4), laboratory studies ($n_{articles}$ = 7), tap water ($n_{articles}$ = 2) and wastewater treatment sludge ($n_{articles}$ = 2).

The type of water treatment process investigated was mostly drinking water treatment ($n_{articles}$ = 19) before and/or after conventional treatment, i.e., coagulation with aluminium chloride, flocculation and/or sedimentation, follow by UV disinfection ($n_{articles}$ = 4) and conventional ozone ($n_{articles}$ = 2) or ozone combined with nanofiltration ($n_{articles}$ = 1), activated carbon ($n_{articles}$ = 1) and combined UV/electrochemical process ($n_{articles}$ = 1). Other investigations explored alternative treatment options, such as magnetic ion exchange (MIEX®) resins ($n_{articles}$ = 3), ultraviolet/persulfate advanced oxidation ($n_{articles}$ = 1), biofiltration with biologically active media ($n_{articles}$ = 1), sunlight irradiation ($n_{articles}$ = 2) and with treatment not specified ($n_{articles}$ = 9). A large number of **DOM sources** were investigated originating mainly from surface water natural DOM ($n_{articles}$ = 31), leaf leachate concentrate ($n_{articles}$ = 5), standard reference materials for humic substances, e.g., Suwannee River Humic Acid ($n_{articles}$ = 5), and algae/bacterial DOM sources, algal blooms and wastewater sludges ($n_{articles}$ = 11). **Chemical disinfection methods**

investigated were chlorination ($n_{articles}$ = 36), chlorine dioxide ($n_{articles}$ = 3), monochloramine ($n_{articles}$ = 4), ozone ($n_{articles}$ = 1) or a combined disinfection method ($n_{articles}$ = 4).

Table S2. Quenching Agent Used in the Selected Articles as a Function of Disinfection Byproduct Classes. Values Expressed as Percent of the Use of each Agent within a DBP Class in the Selected Articles ($n_{articles}$ = 45).

DBP classes	quenching agent									
	ascorbic acid*		sodium thiosulfate		sodium sulfite		ammonium sulfate		ammonium chlorid**	
THMs	29	(+)[a,b]	20	(+)[d]	26	(+)[a,b,d]	3		23	(+)[a]
HAAs	16	(+)[a,b]	21		32	(+)[a], (−)[d]	5		26	(+)[a,d]
HKs	27	(+)[a]	20		20				33	(+)[a,d]
HALs	75	(+)[a]			25	(+)[d]				(−)[c]
X-furanone	100									
HNMs	62	(−)[a]	15		8	(−)[a,b,f]			15	(+)[a,d]
HAMs	100	(+)[b, h]		(+)[h]		(−)[b], (+)[h]				(+)[h]
HANs	41	(+)[a]	14		23	(+)[c] at pH 5.2, (−)[d-g]			23	(+)[a,d]
NAs			100	(+)[e]						
CNX	100	(+)[c] at pH 2–3								

* not suited for inorganic DBPs, ** should be used only when the disinfectant is chlorine. (+) and (−) indicate recommended and not recommended, respectively for use in the selected articles. Reference cited are as follows: a,[26] b,[27] c,[28] d,[24] e,[29] f,[30] g[31], h[32].

ADDITIONAL INFORMATION

Table S3. Summary of Regulatory and Guideline Concentrations in Drinking Water for Currently Regulated Disinfection Byproducts (DBPs).

classes	species	parametric value (µg/L)
regulated DBPs		
THM4s	bromodichloromethane<br>tribromomethane<br>dibromochloromethane<br>trichloromethane	sum of all THM4s = 100 (EU) 80 (US)
HAA5s	monochloroacetic acid<br>dichloroacetic acid<br>trichloroacetic acid<br>monobromoacetic acid<br>dibromoacetic acid	sum of all HAA5s = 60 (EU & US)
unregulated DBPs (guideline)		
HANs	dichloroacetonitrile	20 (WHO) 6 (US)
	dibromoacetonitrile	70 (WHO)
NAs	*N*-nitrosodimethylamine (NDMA)	0.1 (WHO)
	N-nitrosomethylethylamine	0.02 (US)
	N-nitrosodiethylamine	0.008 (US)
	N-nitrosopyrrolidine	0.16 (US)
	N-nitroso-di-n-butylamine	0.063 (US)
	N-nitrosodipropylamine	0.05 (US)
CNX	cyanogen chloride	70 (WHO)

EU = according to the drinking water regulations, European directive 2020/2184,[33] US = according to safe drinking water act, United States Environmental Protection Agency,[34] WHO = according to the World Health Organization.[35]

Table S4. Articles Investigated where Statistically Significant Relationships with PARAFAC Components were Established as a Function of Disinfection Byproduct (DBP) Class and Species.

disinfection byproducts (DBPs)	investigated (%)[a]	relationship established (%)[b]
**trihalomethanes (THMs)**	**89**	**63**
trichloromethane	64	34
bromodichloromethane	56	28
dibromochloromethane	56	28
tribromomethane	51	30
**haloacetic acids (HAAs)**	**51**	**65**
monochloroacetic acid	31	50
dichloroacetic acid	44	40
trichloroacetic acid	40	50
bromochloroacetic acid	20	33
bromodichloroacetic acid	13	17
dibromochloroacetic acid	11	20
monobromoacetic acid	27	33
dibromoacetic acid	33	33
tribromoacetic acid	11	20
**haloketones (HKs)**	**20**	**67**
1,1-dichloro-2-propanone	22	40
1,1,1-trichloro-2-propanone	27	42
**haloacetaldehydes (HALs)**	**16**	**71**
2,2,2-trichloroethane-1,1-diol	20	67
**halogenated furanones (X-furanones)**	**2**	**100**
4-chloro-3-dichloromethyl-2H-furan-5-one	2	100
**iodinated THMs (I-THMs)**		
triodomethane	2	0
bromochloroiodomethane	22	40
bromodiiodomethane	29	46
chlorodiiodomethane	33	60
dichloroiodomethane	38	53
dibromoiodomethane	31	43
**Other carbonaceous DBPs (C-DBPs)**	24	36
2,3-dichloro-4-oxobut-2-enoic acid	20	33
**halonitromethanes (HNMs)**	**4**	**50**
trichloronitromethane	2	0
**haloacetonitriles (HANs)**	**50**	**18**
dichloroacetonitrile	24	74
trichloroacetonitrile	36	31
bromochloroacetonitrile	31	43
dibromoacetonitrile	36	25
***N*-nitrosamines (NAs)**	**4**	**50**
N-nitrosodibutylamine	4	0
N-nitrosodiethylamine	4	0
N-nitrosodimethylamine	2	100
N-nitrosodiphenylamine	2	100
N-nitrosodipropylamine	2	100
N-nitrosomethylethylamine	2	100
N-nitrosomorpholine	2	100
N-nitrosopyrrolidine	2	100
N-nitrospiperidine	2	100
**cyanide (CNX)**	**2**	**100**
cyanogen chloride	2	100

DBP classes are highlighted in bold. [a]Proportion of articles ($n_{articles}$ = 45) where PARAFAC components and DBP formation potential relationships were investigated, [b]proportion where statistically significant linear relationships (moderate or stronger e.g., $R^2 \geq 0.5$) were reported.

SUPPORTING REFERENCES

1. Coble, P. G., Characterization of marine and terrestrial DOM in seawater using excitation–emission matrix spectroscopy. *Mar. Chem.* **1996,** *51*, (4), 325–346.

2. Coble, P. G., Marine optical biogeochemistry: The chemistry of ocean color. *Chem. Rev.* **2007,** *107*, (2), 402–418.

3. Stedmon, C. A.; Markager, S.; Bro, R., Tracing dissolved organic matter in aquatic environments using a new approach to fluorescence spectroscopy. *Mar. Chem.* **2003,** *82*, (3–4), 239–254.

4. Chen, W.; Westerhoff, P.; Leenheer, J. A.; Booksh, K., Fluorescence excitation−emission matrix regional integration to quantify spectra for dissolved organic matter. *Environ. Sci. Technol.* **2003,** *37*, (24), 5701–5710.

5. Murphy, K. R.; Stedmon, C. A.; Waite, T. D.; Ruiz, G. M., Distinguishing between terrestrial and autochthonous organic matter sources in marine environments using fluorescence spectroscopy. *Mar. Chem.* **2008,** *108*, (1–2), 40–58.

6. Cory, R. M.; Miller, M. P.; McKnight, D. M.; Guerard, J. J.; Miller, P. L., Effect of instrument-specific response on the analysis of fulvic acid fluorescence spectra. *Limnol. Oceanogr. Methods* **2010,** *8*, (2), 67–78.

7. Kasparek, A.; Smyk, B., A new approach to the old problem: Inner filter effect type I and II in fluorescence. *Spectrochim. Acta, Pt. A: Mol. Biomol. Spectrosc.* **2018,** *198*, 297–303.

8. Gauthier, T. D.; Shane, E. C.; Guerin, W. F.; Seitz, W. R.; Grant, C. L., Fluorescence quenching method for determining equilibrium constants for polycyclic aromatic hydrocarbons binding to dissolved humic materials. *Environ. Sci. Technol.* **1986,** *20*, (11), 1162–1166.

9. Kothawala, D. N.; Murphy, K. R.; Stedmon, C. A.; Weyhenmeyer, G. A.; Tranvik, L. J., Inner filter correction of dissolved organic matter fluorescence. *Limnol. Oceanogr. Methods* **2013,** *11*, (12), 616–630.

10. Ohno, T., Fluorescence inner-filtering correction for determining the humification index of dissolved organic matter. *Environ. Sci. Technol.* **2002,** *36*, (4), 742–746.

11. Mobed, J. J.; Hemmingsen, S. L.; Autry, J. L.; McGown, L. B., Fluorescence characterization of IHSS humic substances: Total luminescence spectra with absorbance correction. *Environ. Sci. Technol.* **1996,** *30*, (10), 3061–3065.

12. Lawaetz, A. J.; Stedmon, C. A., Fluorescence intensity calibration using the Raman scatter peak of water. *Appl. Spectrosc.* **2009,** *63*, (8), 936–940.

13. Stedmon, C. A.; Bro, R., Characterizing dissolved organic matter fluorescence with parallel factor analysis: A tutorial. *Limnol. Oceanogr. Methods* **2008,** *6*, 572–579.

14. Murphy, K. R.; Stedmon, C. A.; Graeber, D.; Bro, R., Fluorescence spectroscopy and multi-way techniques. PARAFAC. *Anal. Methods* **2013,** *5*, (23), 6557–6566.

15. Andersson, C. A.; Bro, R., The N-way Toolbox for MATLAB. *Chemometrics Intellig. Lab. Syst.* **2000,** *52*, (1), 1–4.

16. Mash, C. A.; Winston, B. A.; Meints Ii, D. A.; Pifer, A. D.; Scott, J. T.; Zhang, W.; Fairey, J. L., Assessing trichloromethane formation and control in algal-stimulated waters amended with nitrogen and phosphorus. *Environ. Sci. Process. Impacts* **2014,** *16*, (6), 1290–1299.

17. R Core Team. R: A language and environment for statistical computing. R Foundation for Statistical Computing. http://www.R-project.org (Access on 2021).

18. Pucher, M.; Wünsch, U.; Weigelhofer, G.; Murphy, K.; Hein, T.; Graeber, D., staRdom: Versatile software for analyzing spectroscopic data of dissolved organic matter in R. *Water* **2019,** *11*, (11), 2366.

19. Helwig, N. E. multiway: Component models for multi-way data. https://CRAN.R-project.org/package=multiway (Access on 24 June 2022).

20. Massicotte, P. eemR: Tools for pre-processing emission-excitation-matrix (EEM) fluorescence data. https://CRAN.R-project.org/package=eemR (Access on 24 June 2022).

21. Rosario-Ortiz, F. L.; Korak, J. A., Oversimplification of dissolved organic matter fluorescence analysis: Potential pitfalls of current methods. *Environ. Sci. Technol.* **2017,** *51*, (2), 759–761.

22. Murphy, K. R.; Hambly, A.; Singh, S.; Henderson, R. K.; Baker, A.; Stuetz, R.; Khan, S. J., Organic matter fluorescence in municipal water recycling schemes: Toward a unified PARAFAC model. *Environ. Sci. Technol.* **2011,** *45*, (7), 2909–2916.

23. Bosco, M. V.; Garrido, M.; Larrechi, M. S., Determination of phenol in the presence of its principal degradation products in water during a TiO2-photocatalytic degradation process by three-dimensional excitation–emission matrix fluorescence and parallel factor analysis. *Anal. Chim. Acta* **2006,** *559*, (2), 240–247.

24. Baird, R. B.; Eaton, A. D.; Rice, E. W., *Standard Methods for the Examination of Water and Wastewater*. 23 ed.; American Public Health Association, American Water Works Association, and Water Environment Federation: 2017; p 1368.

25. Summers, R. S.; Hooper, S. M.; Shukairy, H. M.; Solarik, G.; Owen, D., Assessing DBP yield: Uniform formation conditions. *J. - Am. Water Works Assoc.* **1996,** *88*, (6), 80–93.

26. Kristiana, I.; Lethorn, A.; Joll, C.; Heitz, A., To add or not to add: The use of quenching agents for the analysis of disinfection by-products in water samples. *Water Res.* **2014,** *59*, 90–98.

27. Moore, N.; Ebrahimi, S.; Zhu, Y.; Wang, C.; Hofmann, R.; Andrews, S., A comparison of sodium sulfite, ammonium chloride, and ascorbic acid for quenching chlorine prior to disinfection byproduct analysis. *Water Supply* **2021,** *21*, (5), 2313–2323.

28. Diehl, A. C.; Speitel Jr., G. E.; Symons, J. M.; Krasner, S. W.; Hwang, C. J.; Barrett, S. E., DBP formation during chloramination. *J. - Am. Water Works Assoc.* **2000,** *92*, (6), 76–90.

29. Spahr, S.; Cirpka, O. A.; von Gunten, U.; Hofstetter, T. B., Formation of *N*-nitrosodimethylamine during chloramination of secondary and tertiary amines: Role of molecular oxygen and radical intermediates. *Environ. Sci. Technol.* **2017,** *51*, (1), 280–290.

30. Croué, J. P.; Reckhow, D. A., Destruction of chlorination byproducts with sulfite. *Environ. Sci. Technol.* **1989,** *23*, (11), 1412–1419.

31. Trehy, M. L.; Bieber, T. I., Detection, identification and quantitative analysis of dihaloacetonitriles in chlorinated natural waters. In *Advances in the Identification & Analysis of Organic Pollutants in Water*, Keith, L. H., Ed. Ann Arbor Science: 1981; Vol. 2, p 1192.

32. Ding, S.; Chu, W.; Krasner, S. W.; Yu, Y.; Fang, C.; Xu, B.; Gao, N., The stability of chlorinated, brominated, and iodinated haloacetamides in drinking water. *Water Res.* **2018,** *142*, 490–500.

33. The European Parliament and the Council, Directive (EU) 2020/2184: The quality of water intended for human consumption (recast) *OJEU* **2020,** *63*, (L 435), 1–62.

34. Environmental Protection Agency (EPA), National primary drinking water regulations: Stage 2 disinfectants and disinfection byproducts rules. *Federal Register* **2006,** *71*, (2).

35. WHO *Guidelines for drinking-water quality, fourth edition*; World Health Organization: Geneva, 2017.

36. Zhong, S.; Zhang, K.; Bagheri, M.; Burken, J. G.; Gu, A.; Li, B.; Ma, X.; Marrone, B. L.; Ren, Z. J.; Schrier, J.; Shi, W.; Tan, H.; Wang, T.; Wang, X.; Wong, B. M.; Xiao, X.; Yu, X.; Zhu, J. J.; Zhang, H., Machine learning: New ideas and tools in environmental science and engineering. *Environ. Sci. Technol.* **2021,** *55*, (19), 12741–12754.

37. Baldwin, D. S.; Valo, W., Exploring the relationship between the optical properties of water and the quality and quantity of dissolved organic carbon in aquatic ecosystems: strong correlations do not always mean strong predictive power. *Environ. Sci. Process. Impacts* **2015,** *17*, (3), 619–630.
