# Supplementary material for: Fluorescent Dissolved Organic Matter Components as Surrogates for Disinfection Byproduct Formation in Drinking Water: A Critical Review
Source: ACS ES T Water. 2023 Jun 12;3(8):1997–2008. doi: 10.1021/acsestwater.2c00583 (PMC10425960; doi:10.1021/acsestwater.2c00583)
Supplement: Supplementary file 2 — ew2c00583_si_002.zip [file ew2c00583_si_002.zip › METADATA_extracted data.pdf]

META DATA – Extracted data from the 45 selected papers used for the paper
”Fluorescent Dissolved Organic Matter Components as Surrogates for Disinfection Byproduct Formation in Drinking Water: A Critical Review”

Contents

GENERAL INFORMATION 1
GENERAL DATA INFORMATION 2
HEADER INFORMATION 2
REFERENCES OF THE SELECTED PAPERS 5

GENERAL INFORMATION

Project: PRODOM - Proactive Optical Monitoring of Catchment Dissolved Organic Matter for Drinking Water Source Protection

Version: 8 February 2023

Authors: Elena Fernandez-Pascual, Boris Droz,* Jean O’Dwyer, Connie O’Driscoll, Emma H. Goslan, Simon Harrison, John Weatherill*

Corresponding author: John Weatherill (PI) and Boris Droz

School of Biological, Earth and Environmental Sciences, University College Cork, Cork, Ireland

e-mail: john.weatherill@ucc.ie and bodroz@bluewin.ch

Date of paper selected: 2009 to 2022

Content: Extracted data from the 45 selected papers used in this review. Additional information about the article selection criteria is provide in the Supporting Information (SI) of the associated paper below.

Associated paper:

Fernandez-Pascual, E.; Droz, B.; O’Dwyer, J.; O’Driscoll, C.; Goslan, E. H.; Harrison, S.; Weatherill, J., Fluorescent dissolved organic matter components as surrogates for disinfection byproduct formation in drinking water treatment: A critical review.

Funding: Irish Environmental Protection Agency (Grant No. 2019-W-MS43) as part of the EPA Research Program 2021-2030

License: Creative Commons Attribution v4.0 International (CC BY 4.0)

GENERAL DATA INFORMATION

The data file consists of all data extracted from original paper listed in the references section below. The files are set up read to analysis them in R (https://cran.r-project.org/) and open it using the command:

```
dat <- read.table(file.choose(),header=TRUE, sep="\t", dec=".")
```

NA for **N**on **A**pplicable has been used when the data was not provided in the original articles

HEADER DESCRIPTION

n: unique number for each article

water.source: gave the type of water used in the experiment. synthetic water means water made from pur salt and ultrapure water.

water.treatment.process

If water treatment processes was not provide in the original article, the facilities processes was find out in internet on the water supplier webpage

powdered activated carbon (PAC)

granular activated carbon (GAC)

silver-impregnated activated carbon (SIAC)

Ultraviolet (UV)

potassium permanganate (KMnO4)

ozone (O3)

samples.anal: basic info about the sample

dom.source: nature, origin of the DOM

con.dom_ppmc: dissolved organic matter concentration in mg C per L

water.chara: characterization provide about the water in the articles

dbpfp: disinfection byproduct formation potential investigation in the laboratory: yes or no

each field begin with dbpfp is related to the condition during the dbpfp test.

dbpfp.std.method

"reference method" for DBP-formation potential:

5710B: Baird, R. B.; Eaton, A. D.; Rice, E. W., *Standard Methods for the Examination of Water and Wastewater*. 23 ed.; American Public Health Association, American Water Works Association, and Water Environment Federation: 2017.

UFC_Summers1996: Summers, R. S.; Hooper, S. M.; Shukairy, H. M.; Solarik, G.; Owen, D., Assessing DBP yield: uniform formation conditions. *Journal American Water Works Association* **1996,** *88*, (6), 80-93.

dbpfp.disinfection.method: chemicals use to make the DBPFP test

NH2Cl monochloramine

HOCl chlorine

ClO2 chlorine dioxide

O3 ozone

UV ultraviolet

dbpfp.dose_ppm: disinfection dose in mg per L

dbpfp.residual_ppm: residual disinfection dose in mg per L

dbpfp.quencher: quencher used after the dbpfp time

dbpfp.time_days: time in days of the dbpfp test

dbpfp.temp_deg: temperature in degree to which the dbpfp test was performed

dbpfp.ph: pH of the test

dbpfp.buffer: used of a buffer agent

dbpfp.light.cond: light condition of the dbpfp test. dark or UV ultraviolet.

The follow field refere to the condition of the excitation–emission matrix data acquisition.

spectro.ex.range_nm: range of excitation acquisition in nm

spectro.ex.inc_nm: increment of the excitation acquisition in nm

spectro.em.range_nm: : range of emission acquisition in nm

spectro.em.inc_nm: increment of the emission acquisition in nm

spectro.scan.speed_nmpermin: scan speed in nm per min

spectro.norm spectro.ph: pH of the solution during the eem acquisition

spectro.anal.method: data treatment use to interrogate eem. PARAFAC, peak intensity (peak picking), area integration

spectro.anal.tools: toolbox or software used in the case of PARAFAC

spectro.ver.tools: version of the toolbox or software

spectro.const.vali: test of constrain and component number validation method used

spectro.scat.peaks: scatter used or removed with the procedure (separate by -)

spectro.inner-filt: inner-filter effect procedure, first author and year reported see in reference for the full citation.

parafac.fluo.type: PARAFAC component by fluorophore type. UV and fluorophore_index are used if ultraviolet or any other fluorescence index parameter is reported respectively.

parafac.comp: number of the component in the original articles.

parafac.ex_nm: max peak excitation value in nm. Secondary, third and next peaks if reported in the original paper are separated by ",".

parafac.em_nm: max peak emission value in nm.

dbp.group: desinfection byproduct grouped using the following nomenclature.

carbonaceous disinfection byproducts (C-DBPs)

trihalomethanes (THMs)

haloacetic acids (HAAs)

haloketones (HKs)

haloacetaldehydes (HALs)

halogenated furanones (X-furanones)

iodinated THMs (I-THMs)

nitrogenous disinfection byproducts (N-DBPs),

halonitromethanes (HNMs)

haloacetonitriles (HANs)

Haloacetamide (HAMs)

N-nitrosamines (NAs)

cyanide (CNX)

DBPs : desinfection byproducts species. following IUPAC name following Pubchem data base (https://pubchem.ncbi.nlm.nih.gov/)

nick name like the following are replace:

bromoform --> tribromomethane

chloralhydrate --> 2,2,2-trichloroethane-1,1-diol

chloropicrin-->Trichloronitromethane

NDMA --> N-nitrosodimethylamine

1,1-dichloroacateone -->1,1-dichloro-2-propanone

mucochloric acid --> 2,3-dichloro-4-oxobut-2-enoic acid

nb.eems: number of excitation–emission matrix considered in the relationship

cor.fit : type of correlation made. linear, best fit, polynomial

slope: negative (neg) or positive (pos) slope

r2: correlation coefficients

$r^2 \geq 0.7$ express a strong linear relationship.

r^2 between 0.7 to 0.5 express a moderate relationship.

$r^2 < 0.5$ a weak relationship and not considered as a robust relationship for a prediction purpose.

p.value: level of significance is harmonized between study for comparison:

p-value= 0.1 not significant

p-value≤0.05 significant

p-value≤0.01 strongly significant

ref: first author name and years of the articles. References list could be found in the reference section of this file.

year

country follow ISO 3166 Alpha-3 code schem (https://www.iban.com/country-codes)

AUS Australia

CAN Canada

CHN China

HRV Croatia

IRL Ireland

KOR South Korea

THA Thailand

TWN Taiwan

USA United States of America

REFERENCES OF THE SELECTED PAPERS

Bahram, M.; Bro, R.; Stedmon, C.; Afkhami, A., Handling of Rayleigh and Raman scatter for PARAFAC modeling of fluorescence data using interpolation. *Journal of Chemometrics* **2006,** *20*, (3–4), 99–105.

Beggs, K. M.; Summers, R. S., Character and chlorine reactivity of dissolved organic matter from a mountain pine beetle impacted watershed. *Environ Sci Technol* **2011,** *45*, (13), 5717–5724.

Beggs, K. M. H.; Summers, R. S.; McKnight, D. M., Characterizing chlorine oxidation of dissolved organic matter and disinfection by-product formation with fluorescence spectroscopy and parallel factor analysis. *J. Geophys. Res.* **2009,** *114*, G04001.

Cory, R. M.; Miller, M. P.; McKnight, D. M.; Guerard, J. J.; Miller, P. L., Effect of instrument-specific response on the analysis of fulvic acid fluorescence spectra. *Limnol. Oceanogr. Methods* **2010,** *8*, (2), 67–78.

Gauthier, T. D.; Shane, E. C.; Guerin, W. F.; Seitz, W. R.; Grant, C. L., Fluorescence quenching method for determining equilibrium constants for polycyclic aromatic hydrocarbons binding to dissolved humic materials. *Environ. Sci. Technol.* **1986,** *20*, (11), 1162–1166.

Granderson, C. W.; Pifer, A. D.; Fairey, J. L., An improved chloroform surrogate for chlorine dioxide-and alum-treated waters. *J. - Am. Water Works Assoc.* **2013,** *105*, (3), E103–E114.

Hidayah, E. N.; Chou, Y. C.; Yeh, H. H., Comparison between HPSEC-OCD and F-EEMs for assessing DBPs formation in water. *J Environ Sci Health A Tox Hazard Subst Environ Eng* **2017,** *52*, (4), 391–402.

Hua, B.; Veum, K.; Yang, J.; Jones, J.; Deng, B., Parallel factor analysis of fluorescence EEM spectra to identify THM precursors in lake waters. *Environ. Monit. Assess.* **2010,** *161*, (1–4), 71–81.

Hua, L.-C.; Cai, P.; Huang, C.; Huang, C., Tracking Br-DBPs and bromine substitution factors by two-stage differential characterization of water matrix and NOM during chlorination. *Sci. Total Environ.* **2021,** *782*, 146836.

Huang, X.; Wang, S.; Zhu, S.; Ye, Z., Spectroscopic characteristics and disinfection byproduct formation during UV-assisted photoelectrochemical degradation of humic acid. *J. Cleaner Prod.* **2022,** 375, 134171.

Ji, G.; Sun, S.; Jia, R.; Liu, J.; Yao, Z.; Wang, M.; Zhao, Q.; Hou, L., Study on the removal of humic acid by ultraviolet/persulfate advanced oxidation technology. *Environ Sci Pollut Res Int* **2020,** *27*, (21), 26079-26090.

Jian, Q.; Boyer, T. H.; Yang, X.; Xia, B.; Yang, X., Characteristics and DBP formation of dissolved organic matter from leachates of fresh and aged leaf litter. *Chemosphere* **2016,** *152*, 335–344.

Johnstone, D.; Sanchez, N.; Miller, C., Parallel factor analysis of excitation–emission matrices to assess drinking water disinfection byproduct formation during a peak formation period. *Environ. Eng. Sci.* **2009,** *26*, 1551–1559.

Jutaporn, P.; Armstrong, M. D.; Coronell, O., Assessment of C-DBP and N-DBP formation potential and its reduction by MIEX(R) DOC and MIEX(R) GOLD resins using fluorescence spectroscopy and parallel factor analysis. *Water Res.* **2020,** *172*, 115460.

Jutaporn, P.; Laolertworakul, W.; Tungsudjawong, K.; Khongnakorn, W.; Leungprasert, S., Parallel factor analysis of fluorescence excitation emissions to identify seasonal and watershed differences in trihalomethane precursors. *Chemosphere* **2021,** *282*, 131061.

Kasparek, A.; Smyk, B., A new approach to the old problem: Inner filter effect type I and II in fluorescence. *Spectrochim Acta A Mol Biomol Spectrosc* **2018,** *198*, 297–303.

Kothawala, D. N.; Murphy, K. R.; Stedmon, C. A.; Weyhenmeyer, G. A.; Tranvik, L. J., Inner filter correction of dissolved organic matter fluorescence. *Limnol. Oceanogr. Methods* **2013,** *11*, (12), 616–630.

Kurajica, L.; Ujevic Bosnjak, M.; Kinsela, A. S.; Stiglic, J.; Waite, T. D.; Capak, K.; Pavlic, Z., Effects of changing supply water quality on drinking water distribution networks: Changes in NOM optical properties, disinfection byproduct formation, and Mn deposition and release. *Sci. Total Environ.* **2021,** *762*, 144159.

Kurajica, L.; Ujevic Bosnjak, M.; Novak Stankov, M.; Kinsela, A. S.; Stiglic, J.; Waite, D. T.; Capak, K., Disinfection by-products in Croatian drinking water supplies with special emphasis on the water supply network in the city of Zagreb. *J Environ Manage* **2020,** *276*, 111360.

Lawaetz, A. J.; Stedmon, C. A., Fluorescence intensity calibration using the Raman scatter peak of water. *Appl. Spectrosc.* **2009,** *63*, (8), 936–940.

Lee, H. S.; Hur, J.; Lee, M. H.; Brogi, S. R.; Kim, T. W.; Shin, H. S., Photochemical release of dissolved organic matter from particulate organic matter: Spectroscopic characteristics and disinfection by-product formation potential. *Chemosphere* **2019,** *235*, 586–595.

Lee, H. S.; Hur, J.; Shin, H. S., Dynamic exchange between particulate and dissolved matter following sequential resuspension of particles from an urban watershed under photo-irradiation. *Environ. Pollut.* **2021,** *283*, 117395.

Lee, M. H.; Ok, Y. S.; Hur, J., Dynamic variations in dissolved organic matter and the precursors of disinfection by-products leached from biochars: Leaching experiments simulating intermittent rain events. *Environ. Pollut.* **2018,** *242*, (Part B), 1912–1920.

Li, L.; Jeon, Y.; Ryu, H.; Santo Domingo, J. W.; Seo, Y., Assessing the chemical compositions and disinfection byproduct formation of biofilms: Application of fluorescence excitation-emission spectroscopy coupled with parallel factor analysis. *Chemosphere* **2020,** *246*, 125745.

Li, L.; Liu, T.; Dong, H.; Wang, Y.; Yang, H.; Qiang, Z., Tracking spatio-temporal dynamics of fluorescence characteristics of Huangpu River, China by parallel factor analysis: Correlation with disinfection by-product precursor and pesticide level variations. *Chemosphere* **2021,** *283*, 131198.

Li, W.; Wang, J.; Xin, H.; Li, T.; Duan, J.; Mulcahy, D., Determination of cost-effective optimum coagulant dosage for removal of disinfection by-product precursors in water treatment based on the theory of elasticity. *Journal of Water Process Engineering* **2022,** *47*, 102782.

Lyon, B. A.; Cory, R. M.; Weinberg, H. S., Changes in dissolved organic matter fluorescence and disinfection byproduct formation from UV and subsequent chlorination/chloramination. *J. Hazard. Mater.* **2014,** *264*, 411–419.

Ma, C.; Xu, H.; Zhang, L.; Pei, H.; Jin, Y., Use of fluorescence excitation-emission matrices coupled with parallel factor analysis to monitor C- and N-DBPs formation in drinking water recovered from cyanobacteria-laden sludge dewatering. *Sci. Total Environ.* **2018,** *640–641*, 609–618.

Maqbool, T.; Zhang, J.; Qin, Y.; Ly, Q. V.; Asif, M. B.; Zhang, X.; Zhang, Z., Seasonal occurrence of *N*-nitrosamines and their association with dissolved organic matter in full-scale drinking water systems: Determination by LC-MS and EEM-PARAFAC. *Water Res.* **2020,** *183*, 116096.

Mash, C. A.; Winston, B. A.; Meints Ii, D. A.; Pifer, A. D.; Scott, J. T.; Zhang, W.; Fairey, J. L., Assessing trichloromethane formation and control in algal-stimulated waters amended with nitrogen and phosphorus. *Environ Sci Process Impacts* **2014,** *16*, (6), 1290–1299.

Mobed, J. J.; Hemmingsen, S. L.; Autry, J. L.; McGown, L. B., Fluorescence characterization of IHSS humic substances: Total luminescence spectra with absorbance correction. *Environ. Sci. Technol.* **1996,** *30*, (10), 3061–3065.

Nguyen, H. V.-M.; Lee, M.-H.; Hur, J.; Schlautman, M. A., Variations in spectroscopic characteristics and disinfection byproduct formation potentials of dissolved organic matter for two contrasting storm events. *J. Hydrol.* **2013,** *481*, 132–142.

O'Driscoll, C.; McGillicuddy, E.; Croot, P.; Bartley, P.; McMyler, J.; Sheahan, J.; Morrison, L., Tracing sources of natural organic matter, trihalomethanes and metals in groundwater from a karst region. *Environ Sci Pollut Res Int* **2020,** *27*, (11), 12587–12600.

Ohno, T., Fluorescence inner-filtering correction for determining the humification index of dissolved organic matter. *Environ. Sci. Technol.* **2002,** *36*, (4), 742–746.

Peleato, N. M.; McKie, M.; Taylor-Edmonds, L.; Andrews, S. A.; Legge, R. L.; Andrews, R. C., Fluorescence spectroscopy for monitoring reduction of natural organic matter and halogenated furanone precursors by biofiltration. *Chemosphere* **2016,** *153*, 155–161.

Pifer, A. D.; Cousins, S. L.; Fairey, J. L., Assessing UV- and fluorescence-based metrics as disinfection byproduct precursor surrogate parameters in a water body influenced by a heavy rainfall event. *Journal of Water Supply: Research and Technology-Aqua* **2014,** *63*, (3), 200–211.

Pifer, A. D.; Fairey, J. L., Improving on SUVA 254 using fluorescence-PARAFAC analysis and asymmetric flow-field flow fractionation for assessing disinfection byproduct formation and control. *Water Res.* **2012,** *46*, (9), 2927–2936.

Pifer, A. D.; Fairey, J. L., Suitability of organic matter surrogates to predict trihalomethane formation in drinking water sources. *Environ. Eng. Sci.* **2014,** *31*, (3), 117–126.

Siddique, M. S.; Xiong, X.; Yang, H.; Maqbool, T.; Graham, N.; Yu, W., Dynamic variations in DOM and DBPs formation potential during surface water treatment by ozonation-nanofiltration: Using spectroscopic indices approach. *Chem. Eng. J.* **2022,** *427*, 132010.

Visentin, F.; Bhartia, S.; Mohseni, M.; Peldszus, S.; Dorner, S.; Barbeau, B., Impact of vacuum UV on natural and algal organic matter from cyanobacterial impacted waters. *Environ. Sci.: Water Res. Technol.* **2020,** *6*, (3), 829–838.

Wang, R.; Wang, T.; Qu, G.; Zhang, Y.; Guo, X.; Jia, H.; Zhu, L., Insights into the underlying mechanisms for integrated inactivation of A. spiroides and depression of disinfection byproducts by plasma oxidation. *Water Res.* **2021,** *196*, 117027.

Wang, Y.; Li, L.; Sun, Z.; Dong, H.; Yu, J.; Qiang, Z., Removal of disinfection by-product precursors in drinking water treatment processes: Is fluorescence parallel factor analysis a promising indicator? *J. Hazard. Mater.* **2021,** *418*, 126298.

Watson, K.; Farre, M. J.; Leusch, F. D. L.; Knight, N., Using fluorescence-parallel factor analysis for assessing disinfection by-product formation and natural organic matter removal efficiency in secondary treated synthetic drinking waters. *Sci. Total Environ.* **2018,** *640-641*, 31–40.

Williams, C. J.; Conrad, D.; Kothawala, D. N.; Baulch, H. M., Selective removal of dissolved organic matter affects the production and speciation of disinfection byproducts. *Sci. Total Environ.* **2019,** *652*, 75–84.

Wu, J.; Ye, J.; Peng, H.; Wu, M.; Shi, W.; Liang, Y.; Liu, W., Solar photolysis of soluble microbial products as precursors of disinfection by-products in surface water. *Chemosphere* **2018,** *201*, 66–76.

Xia, Y.; Lin, Y. L.; Xu, B.; Hu, C. Y.; Gao, Z. C.; Tang, Y. L.; Chu, W. H.; Cao, T. C.; Gao, N. Y., Effect of UV irradiation on iodinated trihalomethane formation during post-chloramination. *Water Res.* **2018,** *147*, 101–111.

Xiong, X.; Siddique, M. S.; Graham, N. J. D.; Yu, W., Towards microplastics contribution for membrane biofouling and disinfection by-products precursors: The effect on microbes. *J. Hazard. Mater.* **2022,** *426*, 127797.

Xu, X.; Kang, J.; Shen, J.; Zhao, S.; Wang, B.; Zhang, X.; Chen, Z., EEM-PARAFAC characterization of dissolved organic matter and its relationship with disinfection by-products formation potential in drinking water sources of northeastern China. *Sci. Total Environ.* **2021,** *774*, 145297.

Yang, L.; Kim, D.; Uzun, H.; Karanfil, T.; Hur, J., Assessing trihalomethanes (THMs) and *N*-nitrosodimethylamine (NDMA) formation potentials in drinking water treatment plants using fluorescence spectroscopy and parallel factor analysis. *Chemosphere* **2015,** *121*, 84–91.

Yang, L.; Hur, J.; Lee, S.; Chang, S. W.; Shin, H. S., Dynamics of dissolved organic matter during four storm events in two forest streams: source, export, and implications for harmful disinfection byproduct formation. *Environ. Sci. Pollut. Res.* ***2015,*** *22, (12), 9173–9183.*

Young, T. R.; Deem, S.; Leslie, J. C.; Salo-Zieman, V.; He, H.; Dodd, M. C., Drivers of disinfection byproduct formation and speciation in small, chlorinated coastal groundwater systems: relative roles of bromide and organic matter, and the need for improved source water characterization and monitoring. *Environ. Sci.: Water Res. Technol.* **2020,** *6*, (12), 3361–3379.

Zepp, R. G.; Sheldon, W. M.; Moran, M. A., Dissolved organic fluorophores in southeastern US coastal waters: correction method for eliminating Rayleigh and Raman scattering peaks in excitation–emission matrices. *Mar. Chem.* **2004,** *89*, (1–4), 15–36.

Zhang, Y. X.; Liang, X. Q.; Hua, G. F.; Li, M. R.; Lin, L. M., Disinfection byproduct precursors in paddy fields under swine manure application: Reactivity, origins and interception. *Agric., Ecosyst. Environ.* **2018,** *256*, 173–183.

Zhang, X.; Shen, J.; Huo, X.; Li, J.; Zhou, Y.; Kang, J.; Chen, Z.; Chu, W.; Zhao, S.; Bi, L.; Xu, X.; Wang, B., Variations of disinfection byproduct precursors through conventional drinking water treatment processes and a real-time monitoring method. *Chemosphere* **2021,** *272*, 129930.
